# Variation and change in pronominal address in 19th and early 20th-century German private letters

**DOI:** 10.1515/jhsl-2023-0036

**Published:** 2025-07-31

**Authors:** Markus Schiegg, Stephan Elspaß

**Affiliations:** University of Fribourg, Fribourg, Switzerland; 27257 Paris Lodron Universität Salzburg, Salzburg, Austria

**Keywords:** German, pronominal address, private letters, 19th and 20th century, invisible**-**hand process

## Abstract

This article for the first time provides a corpus-based investigation of the variation and change in the system of German pronominal forms of address in the 19th and early 20th century. Today’s opposition between formal *Sie* (‘Siezen’) and informal *Du* (‘Duzen’) originates in the 18th century; *Sie* mainly replaced formal forms of address with *Ihr* (‘Ihrzen’) or *Er*/*Sie* (‘Erzen’). The data for our analyses are taken from two corpora of private correspondence – patient letters and emigrant letters – with a total of 2,748 letters by 788 writers. Contrary to previous findings that had mainly been based on grammar book accounts and/or anecdotal evidence, our results show that the use of the formal *Sie* pronoun was still rather common when addressing parents in the second half of the 19th century and even the early 20th century. There was no abrupt collapse of the four-tier system of pronominal address to today’s two-tier system but in some regional non-standard varieties of German a three-tier system continued to be used throughout the 20th century. The addressee, the linguistic context and a writer’s changing emotional state prove to be relevant factors of intra-individual variation. The data also suggest that male writers and experienced writers lead the change from formal *Sie* to informal *Du* in addressing parents. Overall, the observed change in pronominal address can be interpreted as a prototypical invisible-hand process.

## 1 Introduction

The pronominal system of address in use today in German originates in an innovation of the 18th century. Whereas addressing a single person with the pronoun *Du* (2nd person singular) had been the default form in private symmetrical communication (‘language of immediacy’, cf. Koch and Oesterreicher 2012) almost throughout the history of German, the *Sie* form of address (grammatically: 3rd person plural) was established as the dominant form of address for the polite ‘language of distance’ (cf. Simon 2003a: 93, 110–114). According to von Polenz (1999: 383), the binary *Du* versus *Sie* system quickly gained ground in the 19th century.

The present paper focuses on the pronominal form of address in private communication. Deplored by grammarians and philologists at the time, the use of the familiar form *Du* by children addressing their parents is reported to have spread rapidly in the first decades of the 19th century (Besch 1996: 109, 2003: 2617). So far, however, there is only anecdotal evidence for this and other findings. The aim of our paper is to provide a sound corpus-based investigation of the variation and change of pronominal address in 19th and early 20th century private letters, especially in letters to parents, but also to other family members and acquaintances.

After a brief overview of the variation and change of the pronominal address system in the history of German and a critical review of the relevant research literature (Section 2), we will present our data in Section 3. Our study draws on two historical corpora: a corpus of historical patient letters and a corpus of historical emigrant letters, with 2,748 private letters in total. Each letter was annotated with metadata about the individual writers, such as age, gender and writing experience, as well as information about the letters, such as date, addressee(s) and address pronouns used (see Section 4 for our methodology). Section 5 presents the results of our study. It shows and discusses the diachronic developments as well as social and individual factors influencing the variation of address pronouns in the patient letters (see Section 5.1) and the emigrant letters (see Section 5.2). The conclusion in Section 6 evaluates the findings against the background of the more general question of how the overall change can be explained. The results permit a differentiated picture of the development of the present-day pronominal address system in German.

## 2 Pronominal address in the history of German

**Figure 1:**
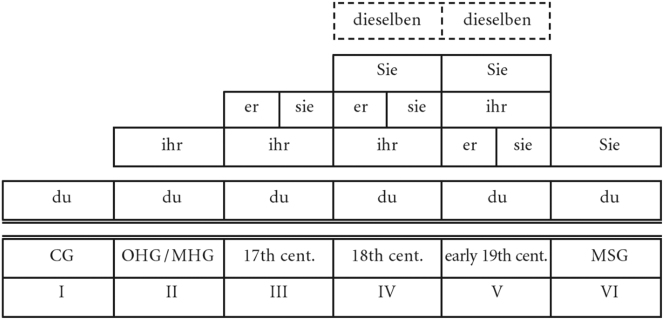
Pronominal paradigms used in reference to a single addressee in the history of German (from Simon 2003b: 86; cf. Simon 2003a: 93).

Like many other European languages, present-day German has a dual pronominal address system, with the older *du* form (2nd person singular for one addressee) and the *ihr* form (2nd person plural for more than one addressee) as the default forms for symmetrical communication in private contexts (T) and the *Sie*-form (grammatically: 3rd person plural; for one or more addressees) as the polite variant (V).1(T) and (V) refer to the so-called T-V distinction, a terminology commonly found in linguistic address research. It is derived from the Latin pronouns *tu* and *vos* and used to classify and distinguish between formal and informal address pronouns. The 19th century is of particular interest for the study of variation and change of the pronominal address system in German, as – according to the research literature – it saw the change from a complex system with four (or five) pronouns to the present-day dual system. Figure 1 gives an overview of the development of the pronominal address system in reference to a single addressee throughout the history of German according to the current state of research.2The spelling of the address pronouns with regard to upper versus lower case initials shifted throughout the history of German. As capital letters were the norm during the period examined in our data, we use this spelling throughout the paper.


Starting from a single form (*du*, stage I), German has developed a complex pronominal address system, which reached its peak in the 18th and early 19th centuries. The only constant throughout the history is the *du* address as the default form for the informal ‘language of proximity’ (or ‘language of immediacy’, in the terms of Koch and Oesterreicher 2012), which already materialises in the earliest sources. In the Middle Ages, the *ihr*-form (originally spelt *ir*; grammatically: 2nd person plural, cf. French *vous*) was added as a form of politeness in communicative formal situations (or ‘language of distance’, cf. ibid.); this two-part system survived until the Early New High German period (stage II). In the 17th century, the system was extended by *Er* (masc.)/*Sie* (fem.; grammatically: 3rd person singular) as polite forms of address (stage III) and finally, in the 18th and early 19th century, by the – even more polite – *Sie* form of address (stages IV and V).3
*Dieselben* was restricted to an anaphoric use with noun phrases like *Ihre Majestät* ‘Your Majesty’ and therefore never fully integrated into the system (cf. Dammel 2017: 211; Simon 2003a: 115–116). In addition, the pronouns *Ihro*/*Dero* as short forms for *Ihro*/*Dero Majestät* were in use in the New High German period but were considered outdated by the mid-19th century (cf. Mentrup 1979: 36). In the corpus of patient letters, these pronouns appear only very rarely in official letters (cf. Schiegg 2022: 228). Like in other languages, the emergence of 2nd and 3rd person plural and 3rd person singular forms of address reflects the application of widespread politeness strategies: the use of plural forms to metaphorically elevate the addressee or the use of indirect address as a face-saving act (Dammel 2017: 207–212; Listen 1999; Simon 2003a: 128–133).

Against this background, it seems particularly worthwhile to conduct an in-depth corpus-based analysis of the developments in the 19th century. The most obvious motivation for such an investigation is that, according to Figure 1, it is the century in which the abrupt change from a four-tier system, which had been built up over more than three centuries, to a two-tier system, which is still valid in the standard language, took place. Dammel (2017: 211) describes the fairly sudden reduction to the dual system with *Sie* as the formal and *du* as the informal address pronoun in the course of the 19th century (stage VI) as a collapse of the ‘overloaded five-tier system’ (“Dieses überladene fünfstufige System”), thus attributing the collapse primarily to language-internal factors, which may be associated with societal changes following the French Revolution (cf. von Polenz 1999: 383). However, the question would then arise as to why this overloaded system was able to function for about a century and a half rather than collapsing earlier.

A further motivation is that we now finally have sufficient data to conduct an in-depth analysis for the 19th century. Such an investigation has only become possible after many new textual sources from this century have been made accessible in the past two decades. In particular, private correspondence from all parts of the population have been made available – for the first time in the history of German – to an extent that allows comprehensive historical sociolinguistic studies (cf. Elspaß 2012a, 2015; Schiegg 2022).

It must be emphasised that the six-stage model outlined above and its sudden reduction accounts for single addressees and the written tradition of German only; the conditions in dialects and other regional varieties of German may even today deviate from this model (cf. Simon 2003b, 2004 for Bavaria). For example, while the use of *Ihr* as formal address had become uncommon in the written language of the early 19th century (cf. Besch 2003: 2616) – and therefore is missing in stage VI in Figure 1 –, it was still used in some regional varieties until the recent past (cf. Besch 2003: 2601; von Polenz 1999: 384). Grober-Glück (1994: 92–93) demonstrates that in the 1930s the use of *Ihr* was the dominant variant for farmhands addressing their employers throughout most parts of Germany, particularly in the west and south-west. Further information on non-standard forms of address comes from dialect grammars and dialect atlases. The dialect atlas of Bavarian Swabia (SBS 2: 173–182), for example, documents the language use in the 1980s and provides evidence for plenty of variation in the address of parents among the older, rural population, born in the first third of the 20th century. While the informants characterise the use of *Sie* as a rare or old variant for addressing parents (one informant used it until around 1945), *Ihrzen* was still far more common. In any case, the loss of the *Ihr* address to single addressees in Bavarian Swabia often only took place in the 20th century.4One of the two authors (M. S.) is able to confirm for his home region that in rural regions of southern Germany the form of address with *Ihr* was still used by elderly speakers until the end of the 20th century. Also, in the western part of German speaking Switzerland, the dialectal form of address is still *Ihr *(cf. Christen et al. 2017: 309). Overall, however, cross-dialect studies and work on diatopic variation in the use of forms of address are lacking so far (cf. Simon 2003a: 125).

Moreover, most previous studies on the system of address in German are based on edited texts from the Middle Ages and the early modern period, and for New High German even only on printed sources. Apart from sporadic observations in the correspondence of the Mozart and Goethe families (cf. Reiffenstein 2009: 69; von Polenz 1999: 384), studies on variation and change in the use of pronominal address forms within family networks are lacking. Thus, hypotheses that are put forward in the research literature and in textbooks on the history of German are often not backed up by empirical corpus-based data of language usage. For example, Besch (2003: 2617), who claims that a relatively rapid change in the pronouns of address used by children towards their parents took place in the first decades of the 19th century, bases his assumptions solely on accounts in various editions of a foreign language grammar of German. Von Polenz (1999: 384) states – without specifying a source – that spouses from the higher social classes mostly used *Sie* (instead of the old *Ihr*) to address each other in the 19th century, as the use of *Du* was considered ‘plebeian’ and ‘obscene’. Findings in patient letters, however, hint at more individuality and variation in actual usage than previously assumed (cf. Schiegg 2022: 227).

To examine variation and change of the German pronominal address system in the 19th and early 20th century more closely, we will use a corpus of patient letters and emigrant letters (see Section 3). The following research questions will guide our investigation:

**RQ1:**

To what extent is the abrupt change in the pronominal address system that is assumed for the 19th century in the research literature reflected in the patient and in the emigrant letters?

**RQ2:**

How do the letter writers vary their use of pronouns of address in relation to the addressees (parents, siblings, spouses, other relatives, friends etc.), and how does this use change during the 19th century?

**RQ3:**

Are social factors, such as writing experience or gender, relevant for the use of address pronouns?

**RQ4:**

Are there forms of intra-individual variation and do writers reflect metalinguistically on their choices of address pronouns?

**RQ5:**

Are there any differences between the two sub-corpora, the patient letters and the emigrant letters respectively?

In the concluding Section 6, we will discuss and try to find explanations for the results on the five research questions.

## 3 Corpora: patient and emigrant letters

Our data come from two historical corpora of patient and emigrant letters from the 19th and early 20th centuries, comprising more than 5,000 letters overall. Extensive metadata is available for most of the writers, so we usually know their hometown, their year of birth, their occupation and their confession, among other things.

The patient letters were taken from the Corpus of Patient Documents (CoPaDocs) that has been compiled by Markus Schiegg’s research group at the Friedrich-Alexander-Universität Erlangen-Nürnberg. It consists of more than 4,000 texts written by patients and their relatives and acquaintances from different German psychiatric hospitals, mainly between 1850 and 1940.5The corpus has been published and can be accessed via DWDS (Digitales Wörterbuch der deutschen Sprache): https://www.dwds.de/d/korpora/copadocs (last accessed 21 February 2024). The analyses for this paper were conducted with a corpus version from August 2022. The letters have survived because they were censored and preserved in the patient files and case books where they can still be found today (Schiegg 2022). The data mostly encompass letters, but also texts of other types, such as autobiographies, notebooks, and poetic texts. Most of the material was retrieved from the southern German psychiatric hospital Kaufbeuren-Irsee, but a few hundred texts also stem from other places in the north (Hamburg) and north-west of Germany (Westphalia). The letters can be differentiated into private letters to family members or friends and official letters to doctors or official institutions. As the present study focuses on pronominal address in private communication, only the 1,767 private letters, written by 432 different people, were selected. 1,424 of these letters by 343 writers are from Kaufbeuren-Irsee, the rest – 343 letters by 89 writers – is of northern German origin.6Some further texts from the southern German institution in Mainkofen are also part of the corpus but were not included in the analysis.


The emigrant letters come from a corpus of 648 letters compiled by Stephan Elspaß in the context of his research on a ‘language history from below’ project (Elspaß 2005, 2015, 2023). About 600 letters were taken from the German Emigrant Letter Collection (‘Deutsche Auswandererbriefsammlung (DABS)’), which comprises a total of 12,000 letters archived in the manuscript department of the Gotha Research Library.7Cf. https://www.auswandererbriefe.de/sammlung.html (last accessed 21 February 2024). The other letters come from various published and unpublished sources (cf. Elspaß 2005: 539–557 for details). Elspaß’s original corpus of 648 documents was expanded over the years by approximately 350 further texts. A large number of these additional letters were newly transcribed by Schiegg’s research group so that the emigrant letter corpus on which the present study is based consists of around 1,000 texts. The letters were written between 1830 and 1938 by emigrants, mostly to North America, to their families at home in the German-speaking areas of the time. There are also a few letters from relatives and friends to the emigrants. As only few texts had to be excluded from the present analysis (one diary, a few official letters, letters written in Dutch etc.), 981 private letters by 356 different writers were considered for this study. Table 1 gives an overview of the composition of the two corpora.

**Table 1:** Composition of the two corpora of private correspondence used in the present study.

	Patient letter corpus	Emigrant letter corpus
Number of letters	1,767	981
Number of writers	432	356
Time span	1850–1949	1830–1962
Regional distribution	80.6 % from southern Germany (Bavarian Swabia), 19.4 % from northern Germany	All German-speaking regions in Germany, Austria and Switzerland

The two corpora show a good diachronic distribution of patient and emigrant letters over the 19th and early 20th centuries, both, however, containing fewer material from the earliest and latest parts of this period. On average, our writers from the psychiatric institutions were born 17 years later than the emigrants.8Birth year emigrants: average 1834, median 1832; birth year patients/acquaintances: average 1851, median 1849. As there is no metadata on this in the documents, we deduced the approximate birth years of acquaintances from the birth year of the patients: (a) acquaintances of the same generation, e.g. siblings/cousins/nieces: same birth year; (b) parents/uncles/aunts: +25 years; (c) children: –25 years.


As usual in historical data, certain asymmetries are unavoidable, which leads to limitations in the scope and the methods applied for the analysis. In the following, we discuss the diatopic, diaphasic and diastratic distribution of the letter material.

The clearest asymmetry in the patient letters is their regional focus on southern Germany, as 80.6 % of the private letters stem from a southern German institution in Kaufbeuren-Irsee. This results from both the good accessibility of the archive where the material is stored today (‘Archiv des Bezirkskrankenhauses Kaufbeuren’) and the research interests of the Erlangen project, which focuses particularly on intra-individual variation. The emigrant letters, in contrast, were collected with the intention to analyse inter-individual regional variation (Elspaß 2005: 68), which leads to a broad regional distribution of the data in the German-speaking areas.

The Erlangen project’s focus on intra-writer variation and in particular addressee design led to a selection of such writers with letters to a number of different addresses (Schiegg 2022: 95). Although the official letters were excluded in the present paper, there is still a high variety of addressees among the private letters, which allows for comparisons of address pronouns in these groups. Emigrant letters, in contrast, were often directed at a number of people at home (Elspaß 2005: 63), which reduces the possibilities of a differentiated analysis of diastratic variation of pronouns used with regard to the addressee.

The two corpora contain texts from writers of diverse social backgrounds. As both corpora were compiled with an interest in the language use of ‘ordinary people’, the majority of the writers are from the lower social classes. This is particularly the case for the emigrant letters, where 317 of the 356 writers (89.0 %) have a low degree of schooling (Elspaß 2005: 46). In our corpus of private patient letters, 292 of the 432 writers (67.6 %) have a manual profession with no necessity to write longer texts on a regular basis so that they can be classified as less-experienced writers (Schiegg 2022: 328). A considerable number of writers in both corpora are women, which is a notable exception among historical corpora as they usually represent predominantly or solely male writing (Nevalainen and Raumolin-Brunberg 2017: 26). The emigrant letter corpus consists of texts by 95 female and 252 male writers,9Three letter writers were classified as both female and male as the letters were written by husband and wife together. Six writers sign with an abbreviated name so that their gender is unknown. i.e. 27.4 % of the writers were female. The corpus of patient letters has 185 female and 245 male writers10Two letter writers (acquaintances) were classified as both female and male as the letters were written by siblings/the grandparents together. (43.0 % female writers). Thus, the patient letter corpus has a better balance of writers’ social backgrounds (their profession and gender) than the emigrant letters, which facilitates comparisons with regard to these variables in the patient letters.

## 4 Methodology

The 2,748 private letters selected for the analysis were annotated manually with regard to metadata about (a) the writers and (b) the letters as well as (c) the address pronouns used in each letter.

(a) For each writer, we annotated their year and decade of birth, regional provenance according to the main dialect areas in the German-speaking countries (cf. Elspaß 2005: 70), gender and profession/education. For the writers of patient letters, we inferred their writing experience from their professions that either involve regular writing practice or consist of manual activities (Schiegg 2022: 328). For acquaintances with an unclear profession (metadata for acquaintances is scarce), we used the same category as for the patient. In the emigrant letters, we referred to the categories basic versus higher educated and thus less experienced versus experienced writers (Elspaß 2005: 46).
(b) Each letter was annotated with the following metadata: the year and decade of writing11In undated letters, the decade of writing can often be inferred from external events, for example from the duration of a hospital stay. Eight patient letters and six emigrant letters were not datable and were therefore excluded from the analysis. and the addressee(s) of the letter. The addressees were placed into six categories: parents, spouses/lovers, siblings, children, relatives, acquaintances/friends. Often we found more than one addressee group in a letter, which led to mixed groups. Due to our research interests in the contextual functions of address and the limited amount of data, we do not restrict our analysis to single addressees, as has been done in previous work (e.g. Simon 2003a).
(c) We are interested both in the morphology of the pronouns and the contextual functions of address (*T* or *V*). Therefore, the letters were also annotated with regard to the address pronouns used and their contextual functions. This is reflected in the glosses to our examples that first explain the morphology and then the function of the pronoun. The following three functional categories are relevant: ‘*Duzen*’ (*T*: informal address; morphology: 2nd person singular/plural), ‘*Siezen*’ (*V*: formal address; morphology: 3rd person plural), ‘*Ihrzen*’ (*V*: formal address; morphology: 2nd person plural). Thereby, we differentiate between one addressee (1) and more than one addressee (>1) (cf. Table 2). When variation appears within the same letter, more than one category of address pronouns was annotated. For example, when different people are addressed in the course of the letter, the address pronouns can change.

**Table 2:** Overview of informal and formal address pronouns in the letter corpus for one addressee and for more than one addressee.

	Informal address (T)	Formal address (V)	
**1 addressee**	Function: ‘*Duzen*’ Form: 2nd person singular pronouns (personal: *Du* NOM, *Deiner* GEN, *Dir* DAT, *Dich* ACC; possessive: *Dein-*)	Function: ‘*Ihrzen*’ Form: 2nd person plural pronouns (personal: *Ihr* NOM, *Euer* GEN, *Euch* DAT, *Euch* ACC; possessive: *Euer-*)	Function: ‘*Siezen*’ Form: 3rd person plural pronouns (personal: *Sie* NOM, *Ihrer* GEN, *Ihnen* DAT, *Sie* ACC; possessive: *Ihr-*)
**>1 addressee**	Function: ‘*Duzen*’ Form: 2nd person plural pronouns (personal: *Ihr* NOM, *Euer* GEN, *Euch* DAT, *Euch* ACC; possessive: *Euer-*)	Function: ‘*Siezen*’ Form: 3rd person plural pronouns (personal: *Sie* NOM, *Ihrer* GEN, *Ihnen* DAT, *Sie* ACC; possessive: *Ihr-*)	

One difficulty in the annotation of the address pronouns is the overlapping of two categories, since 2nd person plural pronouns can be used both as informal address to several persons (T) and as ‘*Ihrzen*’ (V), i.e. a formal address to one person. Only if it is clear from the context that it is just one person who is being addressed with a pronoun of the 2nd person plural, we can safely assign this case to the category ‘*Ihrzen*’. A close reading of the entire letter is always necessary, as the writers sometimes write a letter to one person but then direct wishes or questions to other people as well. For example, our corpus contains two letters by the maidservant Babette B. (kfb-52)12Patient texts are quoted with their name (first name, abbreviated surname), an abbreviation for the former institution (kfb = Kaufbeuren-Irsee) and the file number (here 52) in which the documents concerning a patient are stored. (born 1891), both addressed to her mother. In the letter of 9 April 1911, she uses the formal address of the 3rd person plural (example 1.a), but also 2nd person plural pronouns. In example (1.b), the writer repeats the salutation “Liebe Mutter” (‘Dear Mother’) so that the directly following “Euch” (‘you’-ACC) may first be interpreted as ‘*Ihrzen*’. However, later in the sentence she writes “Euch alle” (‘you all’-ACC), which suggests that Babette B. sends her Easter wishes to several relatives and/or acquaintances at home, who she addresses together informally.

(1)

(a)

**Table:** 

Meinem Versprechen nachzukommen, will ich **Ihnen** sogleich einige Zeilen schreiben […] **Sie** haben versprochen, nach Ostern ganz bestimmt zu kommen.
‘In order to fulfil my promise, I will write **you-DAT-PL-(*1-V-Siezen*)** a few lines immediately […] **You-NOM-PL-(*1-V-Siezen*)** have promised to come after Easter for sure.’

(b)

**Table:** 

Liebe Mutter, möge **Euch** der liebe Gott auf das hohe Fest alles gewähren, was **Euch** alle froh und glücklich machen wird und möge auch der Osterhas recht einlegen u. einkehren.
‘Dear Mother, may the good Lord grant **you-DAT-PL-(*1-V-Ihrzen* or *>1-T-Duzen*)** everything for the high [Easter] feast that will make **you-ACC-PL-(*>1-T-Duzen*)** all happy and joyful and may the Easter Bunny also come and bring many presents.’
(Babette B., kfb-52, maid, letter to mother, 9 April 1911)

The high diversity of the two corpora both with regard to the different text types and the asymmetries in the distribution of the material (see Section 3) led to the decision to analyse the corpora separately and compare the results afterwards. From the imbalances, it also follows that not all possible factors of variation can be analysed in the same way in both corpora. For example, the high variety of addressees in the patient letters allow for more detailed diaphasic analyses than in the emigrant letters.

The imbalances in some dimensions of variation are not necessarily disadvantages but may even facilitate analyses as they reduce the possible factors of variation. For example, the clear dominance of southern German material in the patient letters on the one hand obviates comprehensive diatopic analyses but on the other hand allows a clearer focus on the southern German letters without greater regional imbalances in the use of address pronouns. As a methodological consequence, quantitative analyses of patient letters are conducted only with the southern German material.

The good diachronic distribution of the material makes it possible to trace changes in the use of pronominal address over time using quantitative methods. Consequently, the most important independent variable for the quantitative analyses is time. For a better overview, the diachronic results in Section 5 are therefore presented in 20-year periods, so that the use of different ‘writer generations’ can be compared. At the same time, we need to consider the high diversity of data of the individuals, ranging from one to more than thirty letters per writer (on average: 4.2 letters per patient vs. 2.8 letters per emigrant). Qualitative analyses of intra-individual variation will be of particular interest with authors of multiple letters, as they can provide more detailed insights into the individual language use of the writers to different addressees and over time. The data thus makes it possible to analyse both variation and change in pronominal address.

## 5 Results of the corpus analyses

### 5.1 Pronominal address in patient letters

In the following, we present the results of our analyses of pronominal address in the patient letters. As described in the previous section, we focus on the letters from Kaufbeuren-Irsee, drawing on the material from the other institutions for comparison.

#### 5.1.1 Letters to parents: overview on language change

123 of the 343 people from Kaufbeuren-Irsee wrote at least one of their letters to their parents. Four writers do not use any address pronouns. 54 use formal address pronouns (‘*Siezen*’), while 71 use informal ones (‘*Duzen*’). Some of the writers use both formal and informal pronouns, so they were placed in both categories. Formal address with the 2nd person plural (‘*Ihrzen*’) is quite rare in the letters, as only twelve writers use this variant – five write only *Ihr*, seven use both *Ihr* and *Sie* or *Du* in the same letter.

In most cases, the 2nd person plural pronoun could be connected with wishes or requests directed to relatives at home (cf. Section 4, example (1.b)). Clear cases of ‘*Ihrzen*’ are, for example, references to contents that can only be attributed to one addressee, such as the mother’s name day in the letter by Theodor H. (kfb-882) (born 1850) (example 2), or letters with clearly separate addressees in different parts, such as a letter by Karl W. (born 1839) (kfb-1217) to his mother and siblings, in which he directs a request for trousers and waistcoats directly to his mother (example 3):

(2)

**Table:** 

Ich wünsche **Euch** zum neuen Jahre u. zu **Eurem** Namenstage Alles erdenkliche u. mögliche Gute
‘I wish **you-DAT-PL-(*1-V-Ihrzen*)** all the best for the new year and for **your-DAT-PL-(*1-V-Ihrzen*)** name day.’
(Theodor H., kfb-882, postal assistant, letter to mother, 5 January 1886)

(3)

**Table:** 

Libe Mutter Seid **ihr** so gut u last mir ein par Sontaghosen u westen machen
‘Dear Mother, would **you-NOM-PL-(*1-V-Ihrzen*)** be so kind as to have a pair of Sunday trousers and waistcoats made for me?’
(Karl W., kfb-1217, shoemaker, letter to mother and siblings, 26 September 1864)

As ‘*Ihrzen*’ was still a common phenomenon in Bavarian Swabia in the 20th century (see Section 2), it comes as no surprise that there is no connection with diachrony in the corpus (birth years 1819 [Katharina D., kfb-667] to 1882 [Josefine S., kfb-2287]). Even in the most recent letters from the 1930s, we found instances of *Ihrzen*.13See for example in a letter by Magdalena R.’s (kfb-2950) niece from 14 July 1936. Social class or gender do not seem to play a role either.

Since the number of occurrences for ‘*Ihrzen*’ are comparatively small, they were not included in the quantitative analyses. Also, we have merged the results for one and more than one addressee, as our focus in the following is on the question whether the different groups of addressees are addressed formally (V) or informally (T).

**Figure 2:**
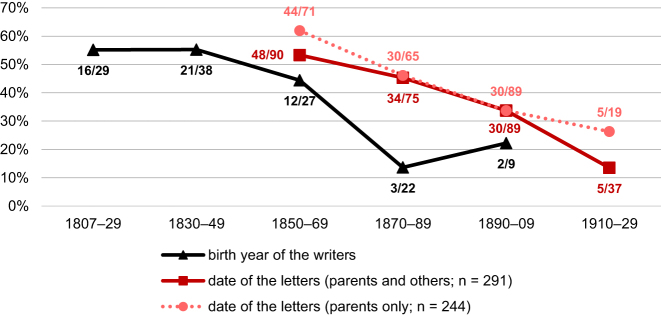
Proportion of Kaufbeuren-Irsee writers and letters with formal *Sie* address to parents over time.

Figure 2 presents the data for the letter writers’ use of formal *Sie* towards their parents. The black graph shows the proportion of writers using the formal address pronoun over time. The writers were grouped according to their birth date, with the earliest writer addressing his parents born in 1807, the latest in 1907. Around 55 % of the writers born in the first half of the 19th century used formal *Sie* when addressing their parents, while there is a slight decrease to 44 % for the years 1850–69 and a sharper decrease to around 20 % by the end of the century.

The psychiatric hospital in Irsee opened in 1849 and the earliest letters in our corpus are from 1850. The other two graphs consider language use in the letters addressed to parents. As there are only two letters addressed to parents from the 1930s in the corpus (both *du*), this period was not included in the figure. The solid red line shows the development of the *Sie* address in all the 291 letters from Kaufbeuren-Irsee in letters to parents. In 47 cases, the letters were also addressed to other relatives such as siblings. These letters were excluded to plot the dotted red line. Again, there is a clear decrease in the use of the *Sie* pronoun over these 80 years. When writers address only their parents, the proportion of the formal address pronoun is higher, particularly in the first (62.0 % vs. 53.3 %) and last period (26.3 % vs. 13.5 %). Contrary to what has been stated in previous research literature (cf. Section 2), the proportion of the formal *Sie* pronoun is still rather high in the second half of the 19th century, and it is, furthermore, commonly used also in the first decades of the 20th century.

#### 5.1.2 Letters to parents: social factors for the use of address pronouns

**Figure 3:**
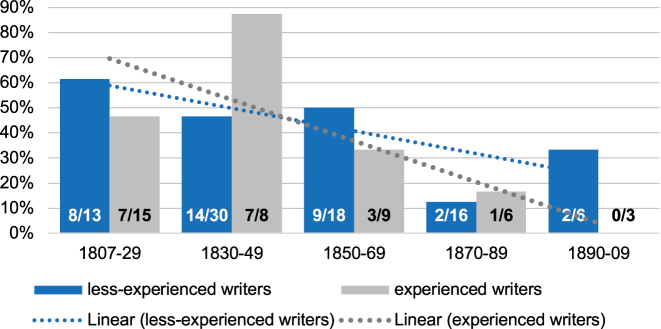
Proportion of Kaufbeuren-Irsee writers using the formal *Sie* address to parents with respect to writing experience (birth years).

Of the 123 people writing to their parents, 80 (65.0 %) came from a background of manual professions such as farmers, craftsmen and maids and were therefore classified as less-experienced writers. 43 (35.0 %) were considered experienced writers as they had worked in professions that involved regular writing such as bookkeepers, merchants, and students. Figure 3 shows the diachronic developments of the *Sie* address in letters to parents divided into these two groups.14As some writers vary in their forms of address (and were thus counted twice) and others do not use any address pronouns at all, the numbers in the graph do not correspond with the number of writers. The rather low number of experienced writers leads to larger fluctuations than in the data of the less-experienced writers. Both groups use the *Sie* address regularly and follow its general decrease over time. However, there seems to be the tendency of a slower decrease in the group of less-experienced writers. Of such writers born in the first half of the 19th century, 51.2 % (22/43) use the formal address (compared to 60.9 % [14/23] of the experienced writers) and of the less experienced writers born in the second half of the 19th century and the beginning of the 20th century (until 1909), 32.5 % (13/40) employ the *Sie* address (compared to 22.2 % [4/18] of the experienced writers). Thus, experienced writers seem to lead this change.

**Figure 4:**
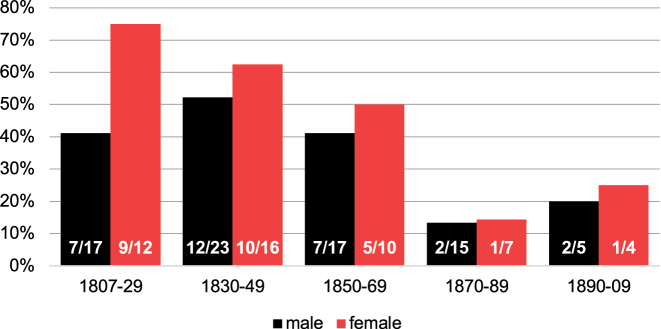
Proportion of Kaufbeuren-Irsee writers using the formal *Sie* address to parents with respect to gender (birth years).

The analysis of use of the *Sie* address with respect to gender shows that both female and male writers regularly use the different forms of address, and that the *Sie* pronoun decreases over time. As Figure 4 illustrates, in any of the five writer generations examined, the proportion of the *Sie* address is higher for females than for males. While again the numbers are rather small, the consistency of these proportions is striking and may either result from women being – or being expected to be – more polite, or it may correlate with the generally lower writing experience of women and the more conservative behaviour of this group with regard to address pronouns. This would conform to our results on writing experience (see Figure 3).

#### 5.1.3 Letters to parents: factors of intra-individual variation

About two thirds of the writers using the *Sie* pronoun (36 out of 55) did this consistently. Interestingly, their average birth year (1837) is 13 years earlier than that of those writers who varied in their language use (1850), which may be an indicator of change. Sometimes, the variation cannot be explained and seems to be random. For example, the corpus contains 12 private letters by the factory worker Viktorie B. (kfb-2853) (born 1864), most of them to her father and her sister. When she addresses her father, she fluctuates between *Sie* and *Du*, sometimes even in the same sentence:

(4)

**Table:** 

muß ich an **Dich**, lieber Vater schreiben, daß **Sie** mich oder meine Lina, sobald wie möglich heimholen!
‘I must write to **you-ACC-SG-(*1-T-Duzen*)**, dear father, that **you-NOM-PL-(*1-V-Siezen*)** bring me or my Lina home as soon as possible!
(Viktorie B., kfb-2853, letter to father and sister, 7 January 1890)

When both parents are addressed, we sometimes find variation between formal and informal plural forms. This can, for example, be observed in a letter by the butcher’s daughter Aloisia S. (kfb-38) (born 1827).

(5)

**Table:** 

Der Allmächige, Wird und soll **Euch** Beite Hier, Und Jensets belohnen Mit Sieben Kronen; Seht **Ihr** in Welch Schöner Lage ich mich befinde. […] um dieses herzli **Sie** Bittend […] Es that mir sehr Leid, mich von **Ihnen** getrennt sehen zu müßen –, ohne Abschied, Und Abbidte getan zu Habn. […] Kön̄en**s** wen̄ Ich nuntr kom̄, leicht vurstelln.
‘The Almighty will and shall reward **you-ACC-PL-(*>1-T-Duzen*)** here and hereafter with seven crowns; **you-NOM-PL-(*>1-T-Duzen*)** see in what a beautiful situation I find myself. […] I sincerely ask **you-ACC-PL-(*>1-V-Siezen*)** for this […] I was very sorry to see myself separated from **you-DAT-PL-(*>1-V-Siezen*)** – without having said goodbye, and having apologised. […] **You-NOM-PL-(*>1-V-Siezen*)** can easily imagine when I come down [i.e. home].
(Aloisia S., kfb-38, letter to parents, around 1850/51)

She starts her letter with the informal pronouns *Euch* (acc.) and *Ihr* (nom.), but later also uses the formal pronouns *Sie* (acc.) and *Ihnen* (dat.). The syntactic contexts for the formal pronouns are rather formal with a present participle verb (“Bittend”) and a complex sentence structure with an infinitive construction and with a tripartite verbal complex (“mich… getrennt sehen zu müßen”). This may have triggered the formal address pronouns. Interestingly, Aloisia S. uses a third form, the enclitic pronoun *s* (for *Sie*; for example, in *Kön̄ens* [can-you-PL-(V)] and *Schrebs.*: abbreviated for *Schre[i]b[en]s* [write-you-PL-(V)]). This form can be characterised as a southern German regional variant (Simon 2004: 358; Werth 2020) of the formal address. However, due to the affinity of regional variants with informal linguistic registers (cf. Schiegg 2022: 335–339) this variant can be classified as less formal than Standard German *Sie* but at the same time more formal than the informal *Ihr* (you-PL-(T)) address. Here, the co-occurring forms are informal dialect variants,15
*nuntr*: *hinunter*; *kom̄*: *komme* (with apocope); *vurstelln*: *vorstellen* (with vowel raising *o* > *u* in prefix and syncope of <e> in last syllable). which again probably triggered the clitic form.

Particularly in formulaic contexts at the beginning of letters, writers sometimes use the formal pronouns. For example, the nailsmith Johann H. (kfb-789) (born 1835) usually addresses his mother with the informal pronoun *Du*, but in a routine formula at the top of his letter from 22 June 1870 he uses *Sie*: “Ich füle mich verpflichted an **Sie** zu Schreiben” (‘I feel obliged to write to **you-ACC-PL-(*1-V-Siezen*)**.’). Similarly, the factory worker Josef R. (kfb-1342) only uses the formal pronoun *Ihnen* in a routine formula at the beginning of most of his letters:

(6)

**Table:** 

Liebe Eltern! Ich teile **ihne** mit, daß soll Vater u. Tante u. Viktor zu uns kom̄e. Und Sei so gut, u. bringt mir gleich mein Werktaganzug
‘Dear parents! I would like to inform **you-DAT-PL-(*>1-V-Siezen*)** that father, aunt, and Viktor are to come to us. And **[you-NOM-SG (*1-T-Duzen*)]** be so good as to bring me my workday suit right away.’
(Josef R., kfb-1342, letter to parents, 24 September 1907)

His verbal forms indicate informal address, but he fluctuates between singular (“Sei”) and plural (“bringt”). His letter in general indicates a lack of competence in written standard German that is, among other phenomena, expressed in such switches. Individual uses of the pronominal system can also be observed in a letter by Jakob H. (kfb-1909) (born 1842), a farmer’s son who wrote one letter to his father on 28 September 1883 and consistently uses *Sie* in the nominative, *Ihnen* in the dative and *Euch* (instead of *Sie*) in the accusative. Thus, there is linguistically conditioned variation between the formal *Sie* and formal *Ihr* address in this text.

Two more experienced writers also show individual uses of the address pronouns. The bookkeeper and salesman Georg B. (kfb-966) (born 1851) consistently addresses his mother and father as individuals with *Du*, but in letters to both of them with *Sie*. Another bookkeeper, Josef B. (kfb-1269) (born 1844), wrote eleven letters to his parents in the years 1876–1878. The nine letters from 1876 are all addressed to both parents and he fluctuates between the formal and informal plural address. In his last letter of this year, he provides a metalinguistic comment on the use of his address pronouns:

(7)

**Table:** 

Sei es mir gestattet den Vater mit “**Du**” anzureden. Ich will einstweilen noch Umgang daran nehmen. Die Mutter würde es beleidigen.
‘May I be allowed to address father as “**you**”**-NOM-SG-(*1-T-Duzen*)**. For the time being, I will avoid it. It would offend mother [if I used it with her].’
(Josef B., kfb-1269, letter to parents, 29 November 1876)

In this quotation, he ponders whether he may be permitted to address his father with *Du*, but wants to avoid addressing his mother with *Du*, as this would offend her. Later in this letter, he addresses his father directly and uses the formal pronoun: “Sei es von **Ihnen** oder von der Mutter, das ist mir gleichbedeutend, lieber Vater.” (‘Be it from **you-DAT-PL-(*1-V-Siezen*)** or from mother, it is all the same to me, dear father.’) In his two later letters, dated 24 December 1877 to his mother and 22 January 1878 to his father, he uses the informal pronouns.

These examples show that there are other factors besides time (or more precisely, date of birth and time of writing) that influence the variation in address pronouns to parents. The following two sections will examine the variation and change of address pronouns to the other addressee groups.

#### 5.1.4 Letters to siblings, children, spouses and lovers

The writers almost consistently use the informal address pronouns (‘*Duzen*’) when addressing their siblings (179 writers with 504 letters from all psychiatric hospitals) and their own children (58 writers, 187 letters).16Only one letter is to a granddaughter: the patient Josefine S. (kfb-2287) received a letter from her grandparents on 24 May 1906. The corpus contains only one formal address in a letter to children, namely when the shoemaker’s wife Maria V. (kfb-2226) (born 1838) uses a formulaic expression at the beginning of a letter to her children on 24 April 1898: “wie geths **Jhnen**” (‘how are **you-DAT-PL-(*>1-V-Siezen*)**’).

Similarly, in letters to siblings, formal address pronouns appear only sporadically and in specific contexts. This can again occur when writers use formulae, such as in a letter by the maidservant Therese W. (born 1851) to her sister, when she closes with a rhyming couplet:

(8)

**Table:** 

Die Drei Feiertage sind verflosen die wir haben genosen Es wünschet **Ihnen** Prosit Neujahr **Ihre** liebe Schwester Theres.
‘The three holidays that we have enjoyed are gone now. **Your-NOM-PL-(*1-V-Siezen*)** dear sister Therese wishes **you-DAT-PL-(*1-V-Siezen*)** a Happy New Year.’
(Therese W., kfb-2091, letter to sister, 27 December between 1882 and 1895)

Another context in which the use of formal and informal pronouns of address varies are letters to different addressee groups, including one that the writers usually address formally. For example, the seamstress Josefine S. (kfb-2287) (born 1882) changes between formal and informal address when she writes to her siblings and father on 17 February 1909 but addresses her father alone (22 July 1906) with the formal address. A few cases are difficult to explain and may result from general politeness, such as in a letter by the son of a factory owner Karl R. (kfb-659) (born 1831) to his sister (2 July 1856) or an intended broader group of recipients in the factory worker Albert F.’s (kfb-2032) (born 1882) postcard to his brother (undated, around 1905–14). Mental illnesses are also a factor that must always be considered in patient letters and that leads to difficulties in writing, for example when the former infantry soldier Karl F.’s (kfb-2882) (born 1849) mixes informal and formal address pronouns in an undated letter to his brother. A strange contrast between different degrees of formality can also be observed in the clerk Karl D.’s (kfb-1614) letters to his sister. While he writes a cordial letter to her on 10 April 1903 (e.g. the closing formula: “schließe ich mit bestmöglichen Grüßen von **Deinem Dich** liebenden Bruder Carl” ‘I end [my letter] with the best possible greetings from **your-DAT-SG-(*1-T-Duzen*) you-AKK-SG-(*1-T-Duzen*)** loving brother Carl’), his letter three years later on 25 February 1906 is excessively formal (“Es grüßt **Sie** mit freundlicher Hochachtung Karl D.” ‘I greet **you-ACC-PL-(*1-V-Siezen*)** with kind regards, Karl D.’ [surname abbreviated by us]). The writer died in November 1907 and the inconsistencies in his texts could have resulted from his deteriorating health.

Another group of people that is usually addressed with informal address pronouns are spouses or lovers (131 writers with 310 letters from all psychiatric hospitals). Occasional examples of formal address pronouns in letters to spouses may again be explained with multiple addressees, such as in the bookkeeper Georg B.’s (kfb-966) (born 1851) letter to his wife and mother-in-law from January 1894, where he uses the *Sie* pronoun. There is yet another case in which a writer changes his language use over time: The shoemaker Cosmas R. (kfb-2108) (born 1825/26) uses the informal address in two letters to his wife in 1872. Ten years later, in April 1882, he wrote a last, overly formal letter to her, before he died from his paralytic illness in July of the same year. His writing ability is clearly connected to his illness, as shown in Schiegg (2022: 367–371) and may have influenced the degree of formality used in his letters. Another context for using the formal address to her husband are the poetic passages that Maria W. (kfb-1745) (born 1848), a tailor’s wife, employs in her letter on 22 June 1882. She metalinguistically reflects on the use of her address pronouns and explains the formal *Sie* with the estrangement from her husband. She varies with the informal address in her letter and also reflects about the “Du Wort” (‘*Du* word’) that she characterises as faithful, pure, slender and fine:

(9)

**Table:** 

Ach lieber werther Herr Gemahl, wie ich **Sie** titelier allchier, o **Sie** liebe, edle Man̄eszier. Den so wie das Schicksall geschieden hat, so hat auch Das Herz geschieden in stumer Gnad […] thuet leide mir, weil ich **Sie** gnädiger Herr titelier Und gar Euer Gnaden noch größere Zier, komts **Du Wort** mitten drunter hinein, so treu, so rein, so schlank so fein, und gar so treu wie die Altbeyerlein
‘Oh dear esteemed husband, as I address **you-ACC-PL-(*1-V-Siezen*)** here, oh **you-ACC-PL-(*1-V-Siezen*)** dear, noble man. Because as fate has parted us, so has the heart parted in silent grace […] (I) am sorry, for although I address **you-ACC-PL-(*1-V-Siezen*)** “gracious Sir” and even “your Grace” or (use) even greater adornments, the “**you-NOM-SG-(*1-T-Duzen*)**” word comes in sometimes, so faithful, so pure, so slender so fine, and even as faithful as the little people from Old Bavaria [*Altbayern*].’
(Maria W., kfb-1745, letter to husband, 22 June 1882)

Due to the low number of 38 texts (by 16 writers), letters to lovers and fiancées were annotated together with the spouses. Again, we find nearly a consistent use of informal address pronouns but, again, with some exceptions. Karl R. (kfb-659) (born 1831), for instance, the son of a factory owner, who has addressed his sister formally (see above), also uses the formal pronouns in a love letter from 2 November 1856 to his girlfriend. Similarly, the pastry cook Friedrich G. (lip-420) (born 1846), treated in the north-west German psychiatric hospital Lippstadt-Eickelborn, addresses his lover in two letters from 22 March 1869 formally. The maidservant Therese W.’s (kfb-2091) four letters to her ‘beloved’ Johann show variation in the use of address pronouns. In an emotional letter from 27 December 1891, she varies between the *Sie* pronoun, then uses informal verbal forms (imperatives singular: “Sei” ‘be’/“schreibe” ‘write’) and repeats and thus intensifies her request, using the southern German enclitic pronoun *s* (“Schreibens” ‘write’; “habens” ‘have’):

(10)

**Table:** 

Haben **Sie** mich nah als **ihre** Gelibte oder als Freundin! Sei so gut und schreibe mir warum Schreiben**s** mir den ^doch^ einmal, haben**s** nicht mer’s Gorasch Geliebter aber ich bitte **Sie** Darum.
‘**You-NOM-PL-(*1-V-Siezen*)** have me close as **your-ACC-PL-(*1-V-Siezen*)** lover or as girlfriend! Be so kind and write me, why do[n’t] **you-NOM-PL-(*1-V-Siezen*)** write me once, don’t **you-NOM-PL-(*1-V-Siezen*)** have the courage for it anymore, beloved, but I ask **you-ACC-PL-(*1-V-Siezen*)** for it.

A short-term diachronic change can be observed in the merchant Otto D. (ham-18626)17The patient file is stored at Staatsarchiv Hamburg, Bestand 352-8/7 (Staatskrankenanstalt Langenhorn), Patientenakte 18626. (born 1903) from Hamburg in his four letters to his lady friend Gertrud in June 1930. He proposed an engagement with her on the 24th, using informal address pronouns. As he did not receive a reply within the following days (his letters were never sent out), he wrote a short card on the 29th, in which he excuses his directness and withdraws his offer, using formal address pronouns.

#### 5.1.5 Letters to relatives and friends

**Figure 5:**
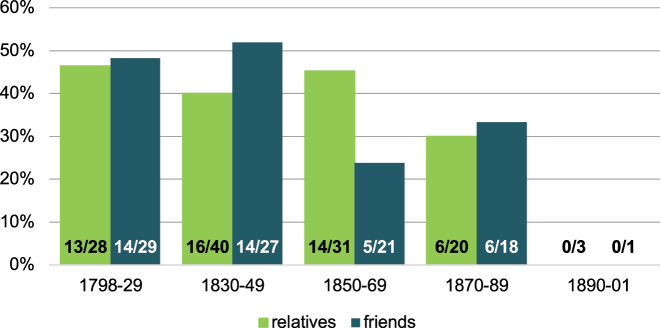
Proportion of Kaufbeuren-Irsee writers using formal *Sie* address with relatives and friends over time.

In letters to relatives and acquaintances, we finally observe more variation in the use of address forms than in those to siblings, children and spouses/lovers, which again allows for a quantitative perspective. Since we do not have sufficient data for a regional/diatopic comparison, we again restrict our analysis to letters from Kaufbeuren-Irsee. The group of relatives contains uncles/aunts, cousins, nieces/nephews, godparents, sisters- and brothers-in-law. Despite the heterogeneity of this group, a diachronic development in the use of formal address pronouns towards relatives is noticeable. 42.6 % (29/68) of the writers born in the first half of the 19th century use formal address pronouns with relatives, which declines to 37.0 % (20/54) for writers born in the second half of that century (see Figure 5, light green bars). In the group of relatives, we find plenty of addressee-dependent variation in the use of address pronouns. Relatives of the same and younger generations are usually addressed informally, while in letters to older relatives, writers sometimes use the formal address. For example, the daughter of a professor, Johanna R. (kfb-343) (born 1823), uses formal *Sie* address for her aunt (9 January 1861) and informal *Du* for her cousin (14 November 1861). Similarly, the farmer’s daughter Maria C. G. (kfb-2827) (born 1845) addresses her uncle formally (31 December 1889), but her nephew informally (3 November 1904). Often, the individual closeness or otherwise to a relative influences the form of address. For example, the day labourer Martin B. (kfb-1621) (born 1832) writes rather informal letters to his “base” – *Base* may refer to a first cousin or other third- or fourth-degree female relative – (15 May 1899; 12 June 1903), using informal address pronouns. In contrast, the letter to his brother-in-law (24 September 1901), who he has not met in person, is highly formal and also contains the formal address pronouns (cf. Schiegg 2022: 303).

The group of friends not only encompasses close friends, but also sometimes neighbours, fellow patients and other unspecified persons. Despite the heterogeneity of this group, there is again a clear diachronic development, from 50.0 % of the Kaufbeuren-Irsee writers born in the first half of the 19th century using the formal address towards acquaintances, to only 27.5 % in the second half (see Figure 5, dark green bars). Again, individual closeness to the addressee can best explain variation in this group. For example, the maidservant Maria E. (kfb-2817) (born 1848) wrote a letter (20 May, year unclear) to “Em̄a Und Mathias”, Emma being an acquaintance from her hometown and Mathias her new husband. When the writer addresses Emma, she uses informal pronouns. As she presumably does not know Emma’s husband personally, she switches to formal pronouns when she directly asks him to pick her up from the psychiatric hospital. It is striking that still in the 1920s and 1930s, some writers differentiate between their address pronouns for friends. For example, the innkeeper and farmer Ludwig F. (kfb-2087) (born 1875) addresses two friends formally (1928/29: *Siezen* and *Ihrzen*) and four other friends and acquaintances informally (1927/28: *Duzen*).

The proportions and general diachronic trends in the use of address pronouns are similar in the groups of relatives and acquaintances and also in comparison to the letters to parents (cf. Figure 2). This confirms the validity of the observations made in the corpus of patient letters.

### 5.2 Pronominal address in emigrant letters

Like the patient letters, emigrant letters show a considerable amount of variation and change in the use of pronominal forms of address. The patterns of intra-individual variation are often similar to those described in the previous sections. For example, the bookkeeper Robert Hager18In contrast to the patient letters, where we abbreviated the surnames for ethical reasons, it is necessary to provide the surnames when referring to emigrant letters because they are archived by surname. (born 1857, emigrated 1880) varies between informal address (*Duzen*) in a letter to his brother Hans in Germany (8 June 1881) and formal address (*Siezen*) in a letter to his mother (23 February 1884). His younger brother Andreas (born 1865, emigrated 1883), who worked as an ordinary sailor, appears to be more progressive, as he uses informal pronouns of address when writing to his mother (3 letters in 1886). As in the patient letters, formal pronouns of address are sometimes restricted to formulaic structures. This comes as no great surprise, as such formulae have already been shown elsewhere to be preservation areas of conservative linguistic – including grammatical – structures (cf. Elspaß 2012b). For example, in a letter to her nephew (18 April 1882), the maidservant Allegonda Look (born 1824) uses the formal address only in the request formula “sind sie so gut” (‘be **you-NOM-PL-(*1-V-Siezen*)** so kind’). Like in the patient letters, cases for *Ihrzen* as formal address are rather uncommon, with only 15 of the writers using this form of address. See example (11), where the writer shifts between *Ihrzen* and *Siezen*:

(11)

**Table:** 

Liebe Onkel. Den Brief, den **Ihr** uns geschrieben habt die haben wir Richtig erhalten und daraus gesehen das **ihr** noch alle Gesund waren. Nun Lieber Frund den ich anspreche als Onnkel jetz will ich **euch** Schreiben wie es uns hier geth den ich habe aus den Brief gesehen **sie** wolten gerne wißen wie es mich mit meine Frau geth.
‘Dear Uncle. We have correctly received the letter **you-NOM-PL-(*1-V-Ihrzen*)** wrote to us and saw from it that **you-NOM-PL-(*>1-T-Duzen*)** were all still healthy. Now dear friend, who I address as uncle, I will now write to **you-DAT-PL-(*1-V-Ihrzen*)** how we are doing here because I have seen from the letter that **you-NOM-PL-(*1-V-Siezen*)** would like to know how I am doing with my wife.’
(Heinrich Elderinck, farmer from Westphalia, letter to uncle, 10 January 1870)

The regional distribution, however, is striking, as only writers from the western part of the German-speaking countries use the *Ihrzen *variant, which confirms Grober-Glück’s (1994: 92f.) observations (cf. Section 2).

Two aspects limit the validity of the analysis of emigrant letters. First, their number in our corpus is far smaller than the number of patient letters – emigrant letters account for only a third of the entire corpus, which makes quantitative analyses difficult. In addition, a larger proportion of emigrant letters has more than one addressee (358 of the 953 that have an identifiable addressee: 37.6 %) than the patient letters (195 of 1743: 11.2 %); this makes it difficult to compare the forms of address used by different groups of addressees. As a consequence, we limit our quantitative analysis to letters in which writers address their parents exclusively, compared to letters in which writers address their parents and other relatives.

**Figure 6:**
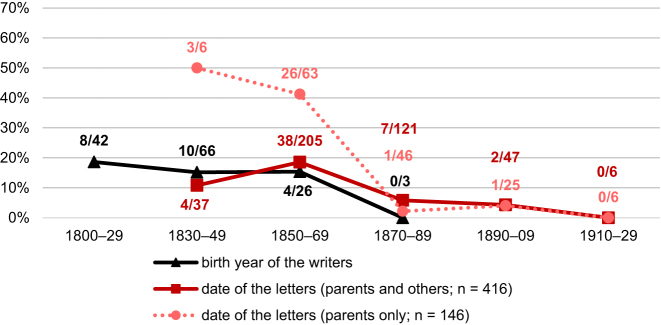
Proportion of emigrant writers and letters with formal *Sie* address to parents over time.

Figure 6 confirms the general trend that the pronominal address with the formal *Sie* variant decreases during the 19th century. Like Figure 2, the graph arranges both the number of writers using the formal address according to their year of birth (black line) and the number of letters with formal address (red lines) on the timeline. Only 24 of the 155 emigrants (15.5 %) use the formal address,19The birth date of two writers who use formal address and of 15 writers who use informal address are unknown, so that they were not included into Figure 6. and even the numbers for those born in the early 19th century are always below 20 %. The dotted red line shows the development of the *Sie* address in letters to parents only, while the solid red line includes these letters and also letters to parents and other relatives. The differences in these two lines are strikingly large in the first two periods analysed (1830–49 and 1850–69). The letters in which writers do not solely address parents – as the prototypical ‘persons of respect’ in the family – show already for these two early periods a largely completed change to the *Du-*form of address. It can therefore be concluded that letters addressed not only to parents but also to addressees for whom the *Du-*form of address had already been established by the beginning of the 19th century (such as siblings, cousins) acted as a gateway for a general *Duzen* towards relatives – including parents.

In comparison to the patient letters, the occurrences of formal address are far fewer in the emigrant letters throughout the data. While only 15.5 % of the emigrants use the formal address (see above), 54 of the 123 writers of patient letters from Kaufbeuren-Irsee (43.9 %) do so, with numbers above 50 % in the first half of the 19th century. Concerning the date of the letters, already in the first period with overlapping data (1850–69), the proportion of the *Sie* address in letters to parents only is far lower in the emigrant letters (41.3 %) than in the patient letters (62.0 %). In addition, the decrease in the use of formal *Sie* address in emigrant letters is very rapid, with only very few instances of formal address from the 1870s onwards, while in 1870–89, 45.3  % of writers of the patient letters still address parents formally.

At this point, the question may arise as to whether the contact of US-emigrants with English and the writers’ increasing familiarity with the simplified system of pronominal address in English could have had an influence on their use of *Du* and *Sie* and, in particular, on the comparatively abrupt change to the *Du-*form of address in letters to their parents. However, this question cannot be answered satisfactorily on the basis of the available data. The emigrant letter corpus consists largely of letters written during or in the first years after emigration, so that the actual period of language contact was relatively short for most of the writers (Elspaß 2005: 71–72). But even in the few cases where series of letters from individual writers over a longer period are available, there is no indication of an influence of language contact on the use of pronominal forms of address. To give just one example, the corpus contains 22 letters written by the four Wesslau siblings (born between 1824 and 1834; see also Helbich and Kamphoefner 2002: 123–136) who emigrated from Jüterbog (North-Eastern Germany) to New York in the 1840s and 1850s. In all these letters, written between 1860 and 1867, they use the formal address when writing to their parents at home. The corpus even provides evidence for the fact that the use of the formal address was taught to the emigrants’ descendants. It contains a letter by the eleven-years old Phyllis Neumayr (born 1895). She was a daughter of the clerk Benno Neumayr, who had emigrated to America in 1881, and his American wife. Phyllis wrote a (fragmentarily preserved) letter to her grandmother in Germany, describing her life in America and using formal address pronouns. For example, she closes her letter as follows:

(12)

**Table:** 

Ich schike **Ihnen** tausend herzliche Grüße von Vater und Mutter besonders aber von Georg und mir. **Ihre Sie** herzlich liebende Enkelin Phyllis Neumayr
‘I send **you-DAT-PL-(*1-V-Siezen*)** a thousand warm greetings from father and mother, but especially from Georg [her brother] and me. **Your-NOM-PL-(*1-V-Siezen*) you-ACC-PL-(*1-V-Siezen*)** loving granddaughter Phyllis Neumayr’
(Phyllis Neumayr, letter to grandmother, 1905)

Benno, in contrast, consistently uses the informal pronouns when writing to his parents, be it to both parents, only to his mother or to parents and siblings (38 letters between 1881–1907).20Unfortunately, the corpus of patient letters contains only very few letters to grandparents so that it does not allow for similar inter-family comparisons.


These observations show that the emigrants on the one hand were rather progressive with the comparatively low numbers of formal address pronouns to their parents. On the other hand, some individuals exhibited conservative behaviour by retaining the linguistic norms acquired in Germany that they – or the German schools – also taught to their children.

## 6 Conclusions

Based on the results presented in Section 5, we can answer our five research questions as follows:

**RQ1:**

To what extent is the abrupt change in the pronominal address system as it is assumed for the 19th century in the research literature reflected in the patient and in the emigrant letters?

**Figure 7:**
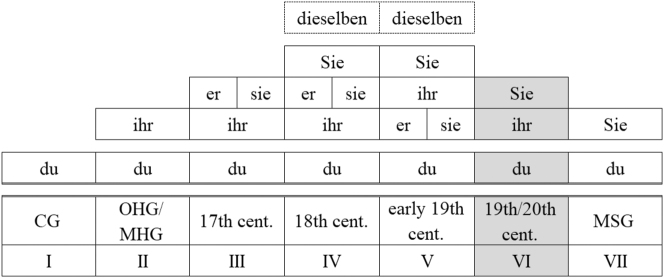
Adapted model of pronominal address in the history of German.

Our two corpora of private correspondence in the 19th century showed some cases of the old *Ihr* as a formal form of address – particularly in the west and the south-west of Germany – even until the 1930s, but no traces of the *Er* and *Sie*-SG variants. In this respect, our results suggest that there was no abrupt change from a four-tier system of pronominal address to a two-tier system in the 19th century, but that, in fact, throughout the 19th century and even in the early 20th century a three-tier system prevailed, so that the ‘overloaded’ four-tier system of the 18th century was only *gradually* dismantled. Dialect data suggest that in some regional non-standard varieties of German, ‘*Ihrzen*’ – alongside ‘*Duzen*’ and ‘*Siezen*’ – and, thus, a three-tier system continued to be used until the end of the 20th century (cf. Section 2). Thus, it does not seem implausible to adopt the following seven-stage model (Figure 7) instead of the six-stage model of the development of the pronominal address system in German presented in Figure 1 – at least for some regions of the German-speaking countries:

**RQ2:**

How do the letter writers vary their use of pronouns of address in relation to the addressees (parents, siblings, spouses, other relatives, friends etc.), and how does this use change in the course of the 19th century?

While there is inter- as well as intra-individual variation, some of it to a considerable extent, overall, the addressee is a significant factor for variation: When addressing parents, writers vary between informal *Du* and the more traditional formal *Sie* (and sometimes old formal *Ihr*), whereas parents consistently address their children and siblings as *Du*. Spouses and lovers are mostly addressed as *Du*. If the letter is addressed to one or more addressees whom the writer normally addresses with *Du* and additionally to one or more addressees normally addressed with *Sie* by the letter writer, both *Sie* and *Du* or *Ihr*-PL can become the default form. In emigrant letters, it appears that letters addressed both to parents (who were traditionally addressed with *Sie*) and siblings or other relatives from the same generation (usually addressed with *Du*) acted as a gateway to an early establishment of the general *Du* address.

**RQ3:**

Are social factors, such as writing experience or gender, relevant for the use of address pronouns?

Although the number of relevant cases is limited, the results from the patient letter corpus suggest that male writers and experienced writers lead the change from formal *Sie* to informal *Du* in addressing parents. There appears to be a correlation between these two innovative groups, i.e. male writers tend to be more experienced. This in turn can be attributed to the fact that – in relation to the total population – men still received a better education than women and, in particular, had better writing skills at the beginning of the 19th century (Elspaß 2005: 86–88).

**RQ4:**

Are there forms of intra-individual variation and do writers reflect metalinguistically on their choices of address pronouns?

Our qualitative analyses identified further factors of intra-individual variation beyond addressee design. We found plenty of evidence for random variation, often resulting from the lack of writing experience. The linguistic context is another relevant factor, as the degree of formality can influence the address form used. Particularly formulaic structures may differ from the predominantly non-formulaic text body of a letter, as formulaic language consists of ready-made linguistic structures for a specific writing situation (cf. Elspaß 2012b: 46). Similarly, address forms in ‘poetic’ passages can deviate from the rest of the text. While emigrant letters did not provide evidence for individual diachronic developments, some patient writers changed their use of address over time. This could be related to their sometimes very emotional state during the writing process or to changing mental conditions of a writer, as deteriorating health could lead to difficulties in writing and thus to inconsistencies in the use of address pronouns. (Perceived) changes in the relationship to an addressee can also lead to an increase of formality and a change of address pronoun. Finally, a switch can also occur as a result of a metalinguistic reflection about how to address parents.

Metalinguistic awareness about address pronouns is only rarely expressed explicitly in the letters. The few cases that appear, however, hint at the writers’ reflection about linguistic variation. This can also be observed in letters by less-experienced writers, such as by a tailor’s wife (see example 9) or in a letter by the farmer’s son Georg W. (kfb-1720) to his parents (5 May 1890). There he comments on the situation in the psychiatric institution, where he is among strangers and exposed to the formal *Sie* address that he had not been used to: “das per **Sie** reden hat mih ganz verführt” (‘Having to use the **you-AKK-PL-(V)** has quite led me astray/got me all confused’, underlined in original).

**RQ5:**

Are there any differences between the two sub-corpora, i.e. the patient letters and the emigrant letters?

The proportion of letters addressed to parents or parents and other relatives with a formal *Sie* address is notably lower in emigrant letters than in patient letters. In the emigrant letters then, the change from ‘*Siezen*’ to ‘*Duzen*’ obviously takes place a good two decades earlier and, thus, more quickly than in the patient letters. Since most of the letters in the emigrant corpus come from the first period of emigration, influences of language contact with English are unlikely. The higher proportion of formal forms of address in the patient letter corpus could, on the one hand, be attributed to rather conservative traditions of politeness in the south-western region from which most (more than 80 %) of the letter writers came; on the other hand, it could also have to do with the fact that the patients’ efforts to be polite and formal in their letters were generally greater, since they expected that this would increase their chances of being discharged from the mental institution. However, both attempts to explain the differences between the two corpora must remain hypothetical.

Earlier in this paper (cf. Section 2), both the research opinion that assumes a sudden change from a five-tier system to a two-tier/dual system in the 19th century and the hypothesis that this change could be explained by an internal factor, i.e. a breakdown of a formerly ultra-complex system of pronominal address, was called into question. In fact, it appears that at least in the north of the German-speaking countries, the rapid change in the pronominal address system can ultimately be traced back to specific measures which were implemented by authorities: After a complaint by a Prussian nobleman in 1764 that he was addressed with *Du* by the tax authorities, the Prussian government inquired as to what forms of address were customary in official letters in the kingdom. Since this survey revealed a regionally very inconsistent usage, the *Du* form of address was henceforth abolished in official letters, so that only the (relatively) new *Sie* form and – until the Stein-Hardenberg reforms (1807–1815) – rarely the old *Ihr* variant were used for formal address (Selchow 1936, quoted after Besch 2003: 2616). Similarly, the use of *Du* and *Er* address to ordinary soldiers, which had been common practice until the beginning of the 19th century, was abolished by decree of the Prussian government in favour of the uniform *Sie* form of address – not least in order to be able to recruit volunteers for the Wars of Liberation (1813–1815); this uniform *Sie* address was also extended to Bavaria and Austria in 1867 (Eckstein 1869: 487, quoted after Besch 2003: 2616–2617). However, it took some time until these changes were adopted in letter writing manuals21For instance, the Bavarian teacher and author Ruckert was still recommending the *Sie* address to parents indiscriminately (Ruckert 1875: 53, 59, 84–86). and, as has been shown in this article, in actual language use.

From a historical sociolinguistic perspective, the relatively rapid change to a two-tier system of pronominal address in the 19th century and the establishment of the *Sie* form of address for formal use could therefore be interpreted as a consequence of prescriptive measures implemented by authorities for formal correspondence, which gradually also affected private correspondence – possibly as a trickle-down effect, with the more experienced writers leading the change. This would constitute an interesting example of a language change ‘from above’, if not a standardisation ‘from above’ (cf. Rutten and Vosters 2021).

However, the expansion of the informal *Du* address in the family sphere, which can be observed parallel to the establishment of the two-tier system of address, particularly through the increasing use of the *Du* form of address towards parents, requires a different explanation. Schröter (2012: 371) discusses the increase in ‘informal’ farewell greetings such as *tschüss* ‘bye, cheers’ in present-day German (cf. Elspaß 2020: 70–72). She argues that this development should not be interpreted as a deliberate choice by speakers to engage in informal linguistic behaviour, but rather as the cumulative result of many individual preferences to express emotional attachment and affection towards addressees (cf. also Dammel 2017: 223). Applied to the pronominal form of address in letters, it is understandable that this desire arose particularly among emigrants, so that the change to the *Du* form of address towards their parents in the homeland can be observed relatively early on in their letters. Ultimately, therefore, an invisible-hand process seems plausible as an explanation for the expansion of the functional scope of the *Du* address, just as Erhardt (2020) sees the development of address pronouns and politeness change, in general, as possible examples of invisible-hand processes (cf. Simon 2003a: 128).

Thematically, our analyses are limited to the variation and change of the pronominal form of address in German and only to two centuries in its recent history. A major concern of our study, however, is to highlight the value of broad-based corpus studies for address research. In particular, corpora from private letters of common writers can – in terms of a linguistic history from below – represent a necessary addition to previous research, which has mainly been based on grammar book accounts and/or anecdotal evidence.

## References

[j_jhsl-2023-0036_ref_001] Besch Werner (1996). *Duzen, Siezen, Titulieren. Zur Anrede im Deutschen heute und gestern*.

[j_jhsl-2023-0036_ref_002] Besch Werner, Besch Werner, Betten Anne, Reichmann Oskar, Sonderegger Stefan (2003). Anredeformen im Deutschen im geschichtlichen Wandel. *Sprachgeschichte. Ein Handbuch zur Geschichte der deutschen Sprache und ihrer Erforschung*.

[j_jhsl-2023-0036_ref_028] Christen Helen, Glaser Elvira, Friedli Matthias (2017). *Kleiner Sprachatlas der deutschen Schweiz*.

[j_jhsl-2023-0036_ref_003] Dammel Antje, Nübling Damaris, Dammel Antje, Duke Janet, Szczepaniak Renata (2017). Anredewandel. *Historische Sprachwissenschaft des Deutschen. Eine Einführung in die Prinzipien des Sprachwandels*.

[j_jhsl-2023-0036_ref_030] Eckstein Friedrich A. (1869). Zur Geschichte der Anrede im Deutschen durch die Fürwörter. *Neue Jahrbücher für Philologie und Pädagogik*.

[j_jhsl-2023-0036_ref_004] Ehrhardt Claus (2020). How to explain diachronic variation of politeness. The example of German pronouns of address. *Lingue e Linguaggi*.

[j_jhsl-2023-0036_ref_005] Elspaß Stephan (2005). *Sprachgeschichte von unten. Untersuchungen zum geschriebenen Alltagsdeutsch im 19. Jahrhundert*.

[j_jhsl-2023-0036_ref_006] Elspaß Stephan, Hernández-Campoy Juan M., Conde-Silvestre Juan C. (2012a). The use of private letters and diaries in sociolinguistic investigation. *The handbook of historical sociolinguistics*.

[j_jhsl-2023-0036_ref_007] Elspaß Stephan, Dossena Marina, Camiciotti Gabriella Del Lungo (2012b). Between linguistic creativity and formulaic restriction. Cross-linguistic perspectives on nineteenth-century century lower class writers’ private letters. *Letter writing in late modern Europe*.

[j_jhsl-2023-0036_ref_008] Elspaß Stephan, Auer Anita, Schreier Daniel, Watts Richard J. (2015). *Letter writing and language change*.

[j_jhsl-2023-0036_ref_009] Elspaß Stephan, Piirainen Elisabeth, Filatkina Natalia, Stumpf Sören, Pfeiffer Christian (2020). Areal variation and change in the phraseology of contemporary German. *Formulaic language and new data*.

[j_jhsl-2023-0036_ref_010] Elspaß Stephan, Lyons Martyn (2023). Common writers in the German-speaking countries from the eighteenth to the twentieth century as agents of a language history from below. *The common writer in modern history*.

[j_jhsl-2023-0036_ref_011] Grober-Glück Gerda (1994). *Die Anrede des Bauern und seiner Frau durch das Gesinde in Deutschland um 1930 unter volkskundlichen und soziolinguistischen Aspekten nach Materialien des Atlas der deutschen Volkskunde*.

[j_jhsl-2023-0036_ref_012] Helbich Wolfgang, Kamphoefner Walter D. (2002). *Deutsche im Amerikanischen Bürgerkrieg: Briefe von Front und Farm 1861–1865*.

[j_jhsl-2023-0036_ref_013] Koch Peter, Oesterreicher Wulf, Lange Claudia, Weber Beatrix, Wolf Göran (2012). *Communicative spaces. Variation, contact, and change. Papers in honour of Ursula Schaefer*.

[j_jhsl-2023-0036_ref_014] Listen Paul (1999). *The emergence of German polite Sie. Cognitive and sociolinguistic parameters*.

[j_jhsl-2023-0036_ref_015] Mentrup Wolfgang, Löffler Heinrich, Pestalozzi Karl, Stern Martin (1979). Großschreibung aus Ehrerbietung – wiewol dieses nicht zur orthographie sondern zur Klugheit … gehöret. *Standard und Dialekt. Studien zur gesprochenen und geschriebenen Gegenwartssprache. Festschrift für Heinz Rupp zum 60. Geburtstag*.

[j_jhsl-2023-0036_ref_016] Nevalainen Terttu, Raumolin-Brunberg Helena (2017). *Historical sociolinguistics. Language change in Tudor and Stuart England*.

[j_jhsl-2023-0036_ref_017] von Polenz Peter (1999). *Deutsche Sprachgeschichte vom Spätmittelalter bis zur Gegenwart. Vol. 3: 19. und 20. Jahrhundert*.

[j_jhsl-2023-0036_ref_018] Reiffenstein Ingo (2009). Sprachvariation im 18. Jahrhundert. Die Briefe der Familie Mozart Teil I. *Zeitschrift für Germanistische Linguistik*.

[j_jhsl-2023-0036_ref_019] Ruckert Alois J. (1875). *Briefsteller für Volks- & Fortbildungs-Schulen. Anleitung zum richtigen Briefschreiben mit mehr als 200 ausgearbeiteten Briefen und Geschäftsaufsätzen*.

[j_jhsl-2023-0036_ref_020] Rutten Gijsbert, Vosters Rik, Ayres-Bennett Wendy, Bellamy John (2021). *The cambridge handbook of language standardization*.

[j_jhsl-2023-0036_ref_021] SBS 2 = König Werner, Feik Christine (2000). *Sprachatlas von Bayerisch-Schwaben. Vol. 2: Wortgeographie I*.

[j_jhsl-2023-0036_ref_022] Schiegg Markus (2022). *Flexible Schreiber in der Sprachgeschichte. Intraindividuelle Variation in Patientenbriefen (1850–1936)*.

[j_jhsl-2023-0036_ref_023] Schröter Juliane, Ernst Peter (2012). „Wenn Menschen auseinandergehn, So sagen sie: auf Wiedersehn“. Zur soziopragmatischen Geschichte eines Abschiedsgrußes im 19. und 20. Jahrhundert. *Historische Pragmatik*.

[j_jhsl-2023-0036_ref_029] Selchow Bogislav von (1936). Du – Er – Ihr – Sie. Anredeformen im Wandel der Zeiten. *Westermanns Monatshefte*.

[j_jhsl-2023-0036_ref_024] Simon Horst J. (2003a). *Für eine grammatische Kategorie ‚Respekt‘ im Deutschen. Synchronie, Diachronie und Typologie des deutschen Anredesystems*.

[j_jhsl-2023-0036_ref_025] Simon Horst J., Taavitsainen Irma, Jucker Andreas H. (2003b). From pragmatics to grammar. Tracing the development of respect in the history of the German pronouns of address. *Diachronic Perspectives on Address Term Systems*.

[j_jhsl-2023-0036_ref_026] Simon Horst J., Gaisbauer Stephan, Scheuringer Hermann (2004). Respekt – die Grammatik der Höflichkeit im Bairischen. *Linzerschnitten. Beiträge zur 8. Bayerisch-österreichischen Dialektologentagung*.

[j_jhsl-2023-0036_ref_027] Werth Alexander, Szczepaniak Renata, Dücker Lisa, Hartmann Stefan (2020). Klisen in frühneuzeitlichen Hexenverhörprotokollen. *Hexenverhörprotokolle als sprachhistorisches Korpus. Fallstudien zur Erschließung der frühneuzeitlichen Schriftsprache*.

